# Combined Proteomic and Metabolomic Profiling of the *Arabidopsis thaliana vps29* Mutant Reveals Pleiotropic Functions of the Retromer in Seed Development

**DOI:** 10.3390/ijms20020362

**Published:** 2019-01-16

**Authors:** Thomas C Durand, Gwendal Cueff, Béatrice Godin, Benoît Valot, Gilles Clément, Thierry Gaude, Loïc Rajjou

**Affiliations:** 1 Laboratoire Reproduction et Développement des Plantes, Université de Lyon, ENS de Lyon, UCB Lyon I, CNRS, INRA, 69342 Lyon, France; thomas.c.durand@gmail.com; 2 Institut Jean-Pierre Bourgin, INRA, AgroParisTech, CNRS, Université Paris-Saclay, 78000 Versailles cedex, France; gwendal.cueff@inra.fr (G.C.); beatrice.godin@inra.fr (B.G.); gilles.clement@inra.fr (G.C.); 3 GQE - Le Moulon, INRA, Univ. Paris-Sud, CNRS, AgroParisTech, Université Paris-Saclay, 91190 Gif-sur-Yvette, France; benoit.valot@univ-fcomte.fr

**Keywords:** retromer, vacuolar protein sorting 29, seed, germination, longevity, proteomics, metabolomics, *Arabidopsis*

## Abstract

The retromer is a multiprotein complex conserved from yeast to humans, which is involved in intracellular protein trafficking and protein recycling. Selection of cargo proteins transported by the retromer depends on the core retromer subunit composed of the three vacuolar protein sorting (VPS) proteins, namely VPS26, VPS29, and VPS35. To gain a better knowledge of the importance of the plant retromer in protein sorting, we carried out a comparative proteomic and metabolomic analysis of *Arabidopsis thaliana* seeds from the wild-type and the null-retromer mutant *vps29*. Here, we report that the retromer mutant displays major alterations in the maturation of seed storage proteins and synthesis of lipid reserves, which are accompanied by severely impaired seed vigor and longevity. We also show that the lack of retromer components is counterbalanced by an increase in proteins involved in intracellular trafficking, notably members of the Ras-related proteins in brain (RAB) family proteins. Our study suggests that loss of the retromer stimulates energy metabolism, affects many metabolic pathways, including that of cell wall biogenesis, and triggers an osmotic stress response, underlining the importance of retromer function in seed biology.

## 1. Introduction

The success of plant reproduction through the production of seeds depends on the proper accumulation of seed storage reserves that will enable germination and early seedling development [1]. In the Brassicaceae family, most of the seed storage compounds are localized in the cotyledons. *Arabidopsis thaliana* seeds store mainly lipid reserves in oil bodies (OBs) and seed storage proteins (SSPs) in specialized organelles, named protein storage vacuoles (PSVs), of the embryo cells [2]. Although the molecular processes sustaining seed reserve accumulation and degradation are still under investigation [1,3,4,5], trafficking pathways taken by SSPs are now broadly understood. The main *Arabidopsis* SSPs, i.e., 2S albumins and 12S globulins, are synthesized in the rough endoplasmic reticulum as precursors. Immediately after they enter the cis-cisternae of the Golgi apparatus, SSPs start their aggregation, which is partly responsible for their subsequent sorting [6]. Once in the trans-Golgi network (TGN), SSPs are transported by electron-dense vesicles to multivesicular bodies (MVBs), where the proteolytic maturation begins [7]. Ultimately, MVBs fuse with PSVs, where SSP maturation and aggregation are completed [8]. Vacuolar sorting receptors (VSRs) are involved in the maturation process as they transport proteolytic enzymes required for SSP maturation from the TGN to the PSV and lytic vacuole. These receptors, such as AtVSR1/AtELP, are recycled to the TGN by a mechanism implicating the retromer complex [9]. The retromer is a phylogenetically conserved protein complex involved in several biological processes by driving the recycling of a variety of membrane receptors [10,11].

First described in yeast [12], the retromer is a pentameric complex composed of two subcomplexes. The small subcomplex is a dimer of sorting nexins (SNXs), which binds to phosphoinositide-containing endosomal membranes. The second subcomplex, also known as the core retromer, consists of vacuolar protein sorting (VPS) proteins, namely VPS26, VPS29, and VPS35 [10]. The retromer complex has been linked to the retrograde transport of several cargo proteins from endosomes to the trans-Golgi network and the recycling of a variety of plasma membrane proteins, and hence is involved in many cellular processes [13,14]. For instance, in metazoan organisms, the retromer complex is implicated in the establishment of cell polarity, morphogenesis, lysosome biogenesis, and different aspects of developmental processes. In humans, alteration of retromer function is associated with a number of pathologies, including Parkinson’s and Alzheimer’s diseases. In *Arabidopsis*, a pioneer study showed that VPS29 plays a key role in cell polarity and organ initiation [15], is required for the efficient sorting of seed storage proteins [9,16], and is required for the biogenesis and degradation of oil bodies [17]. Interestingly, distinct functions for each retromer subcomplex have been described in *Arabidopsis* [16], and an essential role for VPS35 in the assembly of the core retromer to the endosomal membrane has been reported [18]. 

In the present study, we investigated the impact of the *vps29* knockout mutation, which leads to the complete loss of retromer function [15], on seed physiology. Indeed, this null mutation is an opportunity to establish how a nonfunctional retromer affects seed physiology and how molecular networks are reorganized in the mutant seeds. We focused our analysis on dry mature seeds, because at this stage, a full stop in the plant cycle development is achieved. Seed quality is a key parameter for plant dissemination, production, and yield, which are of economic and ecological importance [19,20]. We assessed seed vigor through germination and longevity tests for both *vps29* and Col-0 seeds. In order to get a large picture of the comparison between seeds from *vps29* and Col-0 genotypes, a nontargeted approach was chosen without a priori combining proteomic and metabolomic differential analyses.

The seed proteome was established by both two-dimensional electrophoresis (2DE) gel-based and shotgun proteomics and led to the identification of more than 2000 proteins in dry mature *Arabidopsis* seeds. The 2DE gel-based proteomics has been very successful in studying seed biology [21,22,23,24], plant development, or plant responses to various stresses [25,26]. The quality and quantity of metabolites present in the seed are crucial for its ability to grow a thriving plantlet. In order to explore the *Arabidopsis* seed metabolome, gas chromatography/mass spectrometry (GC/MS)-based metabolic profiling was performed. This metabolome is the output of the highly regulated processes leading to seed formation. Similarly, the proteome of the quiescent dry seed is in a fairly static phase. So, the molecular response to the environment is very subtle at this developmental stage. Hence, the proteome of dry mature seeds might be considered as the final product of the gene expression program during seed development on the mother plant. Therefore, the combined analysis of the proteome and metabolome appears as a pertinent exploratory strategy for understanding the level of involvement of the retromer complex in the plant life cycle. We here describe how disruption of the retromer more globally impacts the metabolism of the organism, using proteomics and metabolomics on *Arabidopsis* seeds as a model system. We reveal that lack of a functional retromer induces severe, unanticipated modifications of the seed proteome associated with major changes in metabolism. 

## 2. Results

### 2.1. Impact of the vps29 Mutation on Seed Physiology

**Figure 1:**
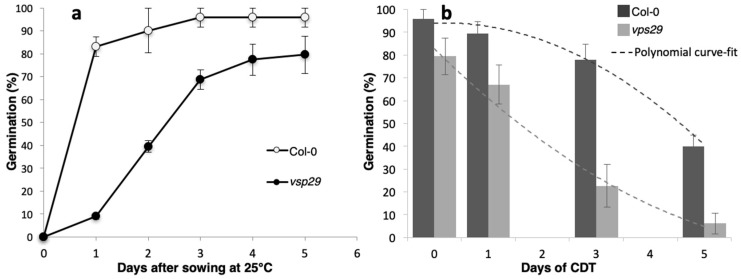
Impact of the loss of retromer function on seed germination and longevity. (**a**) Germination curve of Col-0 and *vps29 Arabidopsis* seeds after cold stratification treatment (5 days at 4 °C in the dark) and subsequent transferral to continuous light at 25 °C. Seed germination (considered as radicle protrusion through the seed coat and the endosperm) was scored daily. White circles represent the germination means for Col-0 seeds. Black circles represent the germination means for *vps29* seeds. The bars represent the standard deviation (±SD) of the mean of three independent experiments. (**b**) Influence of controlled deterioration treatment (CDT) on *Arabidopsis* seed germination. Col-0 and *vps29* seeds were submitted to CDT for different periods of time (0, 1, 3, and 5 days). The graph shows the maximum percentage of germination (Gmax) of deteriorated seeds. It is a representative experiment carried out three times in triplicate. The bars represent the standard deviation (±SD) of the mean of three independent experiments. Dashed lines represent the polynomial curve-fit related to these following equations: Col-0, y = −2.3054x^2^ + 5.4464x + 90.901 with R^2^ = 0.99037; *vps29*, y = 1.5537x^2^ − 26.453x + 107.67 with R^2^ = 0.98204.

Seed germination tests revealed that *vps29* mutation led to a marked decline in seed vigor, immediately affecting the speed, homogeneity, and final rate of *Arabidopsis* seed germination (Figure 1a). Indeed, Col-0 stratified seeds germinated at more than 80% 1 day after sowing (DAS) at 25 °C and reached a maximum percentage of germination (Gmax) close to 100% by 3 DAS. By contrast, *vps29* stratified seeds germinated about 10% on 1 DAS at 25 °C and reached a Gmax close to 80% on 4 DAS. In addition, *vps29* seeds displayed a higher sensitivity to controlled deterioration treatment (CDT), reflecting a lower longevity (Figure 1b). The *vps29* seeds also displayed a smaller thousand seed weight (TSW) than the wild-type seeds (Col-0_TSW_ = 24.8 ± 0.7 mg/1000 seeds; *vps29*_TSW_ = 17.3 ± 0.6 mg/1000 seeds).

### 2.2. Gel-Based Proteomics between Col-0 and vps29 Dry Mature Seeds

**Figure 2:**
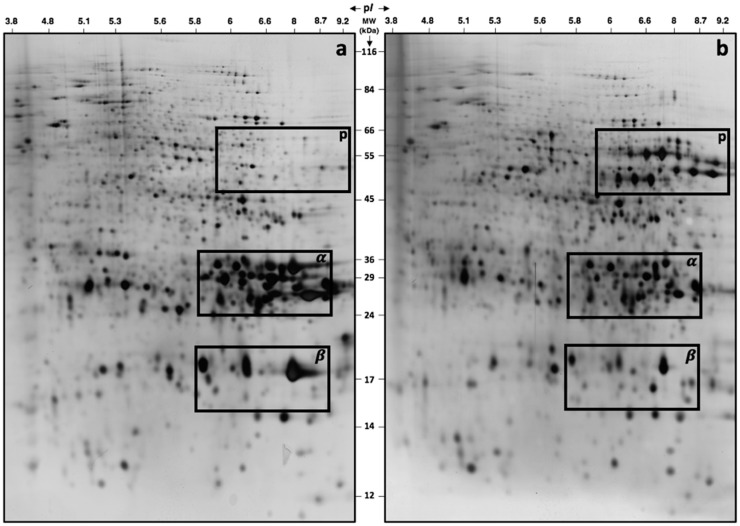
Influence of the retromer *VPS29* mutation on *Arabidopsis* dry seed proteome. An equal amount (150 µg) of total protein extracts was loaded on each gel. (**a**) A representative silver nitrate-stained 2DE gel of total soluble proteins from Col-0 (*Arabidopsis*, accession Columbia-0; wild-type) dry mature seeds. (**b**) A representative silver nitrate-stained 2DE gel of total soluble proteins from *vps29* dry mature seeds. The rectangles highlight the protein clusters of 12S storage protein precursors (p), 12S alpha-cruciferins (α), and 12S beta-cruciferins (β). MW, molecular weight; pI, isoelectric point.

To find out possible clues explaining defects in the seed physiology of the mutant, we investigated the impact of retromer disruption on the seed proteome through 2DE gel-based proteomics. Total soluble proteins from dry mature seeds were separated by 2DE, and protein patterns were characterized by image analysis. Because *vps29* is known to be impaired in storage protein maturation [9], we paid particular attention to changes in the abundance of the major storage proteins, such as 12S cruciferins (Figure 2). In the wild-type seeds, the 2DE analysis detected the three forms of 12S cruciferins [22,27]: the precursor form (shown in frame “p” in Figure 2a,b), α-subunits (shown in frame “α” in Figure 2a,b), and β-subunits (shown in frame “β” in Figure 2a,b). As expected, *vps29* seeds contained a higher abundance and diversity of 12S precursor isoforms and a lower abundance of both α- and β-subunits. *vps29* seed proteome changes were not limited to seed storage proteins and also affected a large number of other proteins. 

### 2.3. Quantitative Shotgun Proteomics between Col-0 and vps29 Dry Mature Seeds

**Figure 3:**
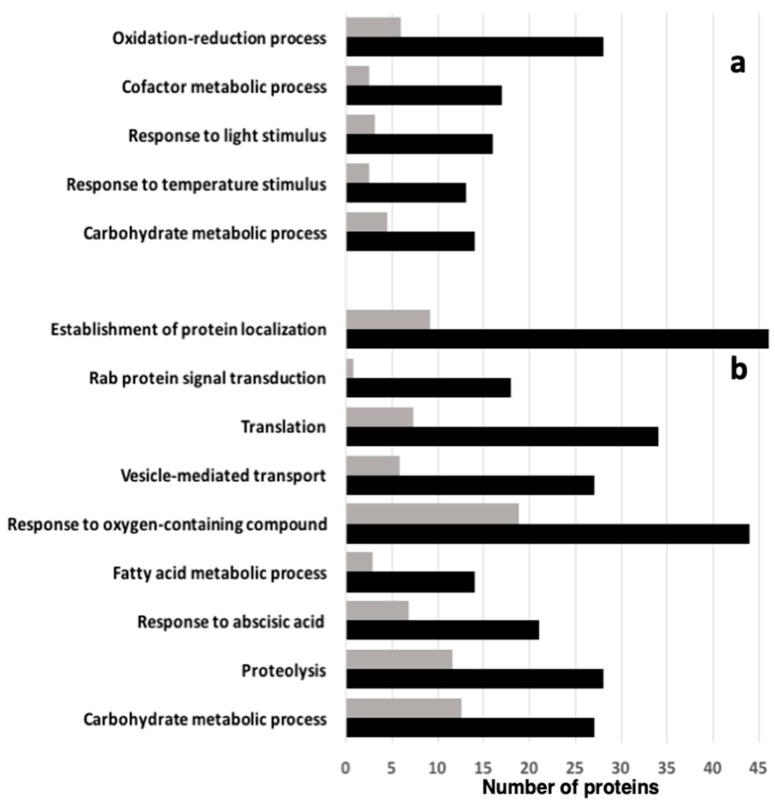
Gene ontology (GO) term enrichment analysis of the selected 442 differentially abundant proteins in *vps29* dry seeds in comparison with Col-0 seeds. (**a**) Overrepresented GO terms for 115 proteins that were up-accumulated in Col-0 dry seeds. (**b**) Overrepresented GO terms for 327 proteins that were up-accumulated in *vps29* dry seeds. Data from PANTHER overrepresentation test (http://www.geneontology.org); *Arabidopsis thaliana* GO database released 1 June 2018. Black bars: observed proteins; grey bars: expected result from the reference *Arabidopsis* genome.

To go further in the comparative analysis of Col-0 and *vsp29* seed proteomes, we opted for a shotgun proteomic approach. The shotgun proteomic approach performed on the Col-0 and *vps29* dry mature seeds resulted in the identification of a total of 2192 protein subgroups (Table S1). Thus, 1963 and 2108 proteins were identified in Col-0 and *vps29* seeds, respectively. Whereas 1879 proteins were shared between the two genotypes, 84 and 229 proteins were detected only in Col-0 or *vps29* seeds, respectively (Table S1). We carried out a differential analysis of Col-0 and *vps29* seed proteomes by spectral counting, and 710 proteins were sorted as differentially abundant (nonparametric Kruskal–Wallis k-samples test, *p* value < 0.05; Table S2). To restrict the analysis to significantly differentially accumulated proteins, we chose first to consider only proteins with an average spectral count above 2 and a fold change above 2 between Col-0 and *vps29* seed proteomes. This allowed us to focus on 442 proteins differentially increased in abundance (i.e., up-accumulated), including 115 proteins in Col-0 and 327 in *vps29* seeds (Table S2). Interestingly, we found that the *vps29* mutant exhibited 2.84-fold more up-accumulated proteins than those decreased in abundance (i.e., down-accumulated) compared with the wild-type. A gene ontology (GO) analysis showed that 208 GO terms were overrepresented among the 442 differentially abundant proteins (Table S3). GO term enrichment in the 115 up-accumulated proteins in Col-0 includes functions such as oxidation–reduction processes, cofactor metabolic processes, responses to temperature and light stimulus, and carbohydrate metabolic processes (Figure 3a; Table S3). GO terms such as protein localization, Rab protein signal transduction, translation, vesicle-mediated transport, responses to oxygen-containing compounds, proteolysis, responses to abscisic acid, lipid metabolic processes, and carbohydrate metabolic processes were overrepresented in the 327 up-accumulated proteins in the *vps29* dry seed proteome (Figure 3b; Table S3).

### 2.4. Quantitative GC/MS-Based Metabolic Profiling of *Arabidopsis* Col-0 and vps29 Dry Mature Seeds

Because the GO term enrichment analysis suggested possible major changes in several metabolic pathways in the mutant seeds, we carried out metabolic profiling of Col-0 and *vps29* dry seeds. The metabolomic approach led to the quantification of 143 metabolites, among which the abundance of 75 were significantly altered in *vps29* seeds compared with Col-0 seeds (nonparametric Kruskal–Wallis k-samples test, *p* value < 0.05; Table S4). Thus, 24 and 52 metabolites were up-accumulated in Col-0 and *vps29* seeds, respectively (Table 1 and Table 2 and Table S4).

## 3. Discussion

### 3.1. Seed Germination and Longevity: An Integrative View Based on vps29 Proteomics and Metabolomics

Previous studies demonstrated that vacuolar sorting receptor (VSR) proteins are associated with germination success and seedling establishment, suggesting that deposition on storage proteins in PSVs during seed development is important for seed vigor [28,29]. Our analysis of the germination potential of *vps29* seeds clearly shows that loss of retromer function has a strong effect on the vigor and longevity of the mutant seeds. This raises the question of the molecular determinants involved in this deficiency.

Our results showed that α-tocopherol as well as proline were more abundant in Col-0 seeds than in *vps29* seeds (by 2.64-fold and 1.79-fold, respectively, Table 1 and Table S4). Previous work documented that a primary function of tocopherols (vitamin E) in seeds is to limit nonenzymatic lipid oxidation during seed storage, germination, and early seedling development, and hence to protect seed viability [30]. As an osmoprotectant, proline also has antioxidant properties and a central role in desiccation tolerance [31]. Proline deficiency in *vps29* seeds is balanced by a significant increase in abundance of other metabolites displaying osmoprotective properties, such as xylitol (26-fold higher, ↗26) and sorbitol (↗22), but also trehalose (↗19.6), myo-inositol (↗3.41), sucrose (↗1.48), and galactinol (↗1.49) (Table 2 and Table S4). 

The content of raffinose and stachyose was not modified, in spite of a more abundant raffinose synthase 4 (At4g01970, ↗2.75). Thus, *vps29* seeds do not display problems with desiccation tolerance, but are mainly affected in terms of longevity and germination vigor. Our proteomic approach also reveals that proteins involved in oxidation–reduction processes and responses to temperature stimulus are overrepresented in Col-0 seeds (Figure 3; Table S3). As seed longevity was assessed by CDT at elevated temperature (40 °C), we conclude that *vps29* seeds are less well-armed to withstand dry storage and maintain their germination potential in the long term. Our combined analysis of the proteome and metabolome of wild-type and *vps29* mutant seeds provides clues on the molecular mechanisms that are associated with the *vps29* seed phenotype and hence may explain it.

### 3.2. The vps29 Mutation Affects Energy Metabolism in Dry Mature Seeds

**Figure 4:**
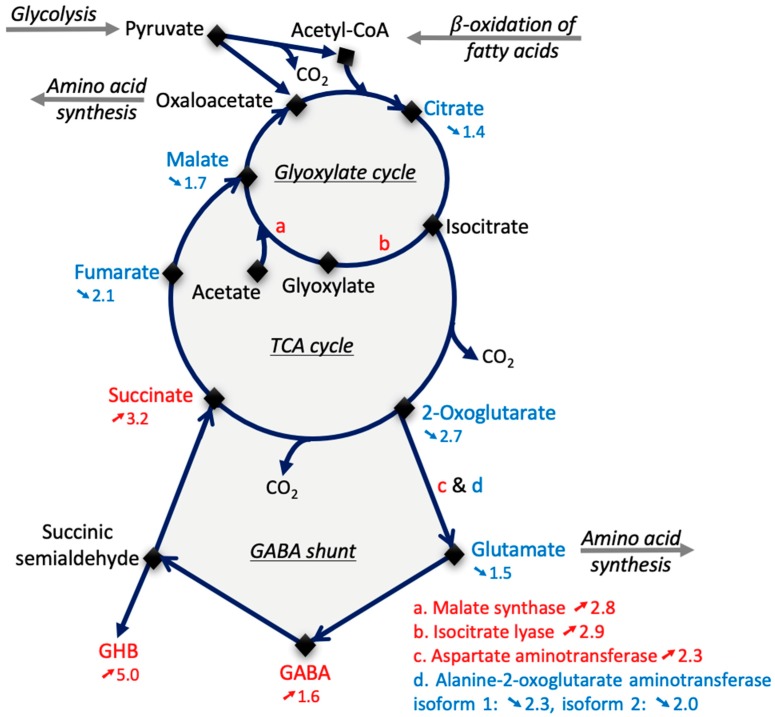
The energy metabolism is altered in *vps29 Arabidopsis thaliana* seeds. Numbers preceeded by an upward or downward arrow indicate the *vps29*/Col-0 positive (in red) or negative (in blue) abundance ratio of the enzymes (see Table S2) or metabolites (see Table 1, Table 2, and Table S4), respectively.

There are several lines of evidence that indicate that energy metabolism is highly modified in *vps29* seeds. Among the metabolites involved in the tricarboxylic acid (TCA) cycle, the levels of several of them were significantly reduced in *vps29* seeds, such as 2-oxoglutarate (also named ketoglutarate, with a 2.7-fold lower ratio, ↘2.7), fumarate (↘2.1), malate (↘1.7), and citrate (↘1.4). By contrast, succinate was more abundant (↗3.2) in *vps29* seeds. The TCA cycle provides precursors for the biosynthesis of certain amino acids through transamination reactions, involving, for example, aspartate or glutamate as amino-group donors. Interestingly, despite the increased abundance of aspartate aminotransferase 2 (At5g19550, ↗2.3), which catalyzes the reversible transfer of the amine group from aspartate to 2-oxoglutarate to produce oxaloacetate and glutamate, the content in glutamate was reduced in *vps29* seeds (↘1.5). This may be simply due to the initial reduced amount of the substrate 2-oxoglutarate (↘2.7) in the mutant, which is likely to be a limiting factor in the reaction. The interconversion between glutamate and 2-oxoglutarate is under the control of other enzymes, the abundance of which were also reduced in *vps29*, such as glyoxylate aminotransferase 2 (At4g39660, ↘2.1), glutamate dehydrogenase 1 (At5g18170, ↘2.1), and two isoforms of alanine-2-oxoglutarate aminotransferase (At1g23310, ↘2.3 and At1g70580, ↘2). These changes in abundance support the hypothesis of an activation of the gamma-aminobutyric acid (GABA) shunt (GABA ↗1.6) in *vps29* seeds (Figure 4). The increased amount of the Ca^2+^-binding EF-hand family protein (At1g18210, ↗10) may be related to the Ca^2+^ dependency of the GABA shunt, which is the route from glutamate (↘1.5) to succinate (↗3.2). The GABA shunt plays a major role in primary carbon and nitrogen metabolism in plants, in close association with the TCA cycle [32,33]. Gamma-hydroxybutyric acid (GHB ↗5) is a derivative of GABA. It is noteworthy that GABA can reduce oxidative damage in stressed plants [34], whereas GHB may cause such damage by inducing lipid peroxidation in animals [35].

The glyoxylate cycle allows the seeds to use the lipid reserves for the production of sugars. The two main enzymes of the glyoxylate cycle are isocitrate lyase (At3g21720), which produces glyoxylate and succinate, and malate synthase (At5g03860). In *vps29* seeds, these two enzymes were similarly up-accumulated, with an increase of 2.9 and 2.8, respectively. This suggests that the glyoxylate cycle is overactivated during the late stages of seed maturation. On the fringe of the TCA and glyoxylate cycles, the metabolism of alanine appears particularly altered. While alanine was more abundant in *vps29* (↗6.5), β-alanine dropped in abundance (↘ 9.1). A possible cause for the β-alanine decrease might be the low abundance of β-ureidopropionase (PYD3, At5g64370, ↘2.4), the enzyme that produces β-alanine. It is noteworthy that increased abundance of alanine is considered as a universal stress signal [36].

Gluconate (↗5.6), which results from the oxidation of glucose, was the third most abundant metabolite in *vps29* seeds relative to GC/MS signal intensity (Table S4). Galactonate (↗1.7) comes from the oxidation of galactose. Along with an increase in the abundance of glycerate (↗3.1), glycerol (↗3), and glycerol-3-Phosphate (↗2.8) and decrease in glucose abundance (↘1.5), this indicates an increase in energy metabolism. The limitation in carbon availability was also reported to promote the GABA shunt [33], which is in accordance with the hypothesis that the GABA shunt is activated in the mutant. 

Other lines of evidence in favor of an energy burst in the mutant are, for instance, the marked increase in abundance of several H^+^-ATPases (At2g18960, ↗23.5; At4g27500, ↗15; At1g78920, ↗9; At4g39080, ↗6.5; At2g20140, ↗6.5; and At1g07670, specific to *vps29* seeds). The increase of the energy metabolism appears to be essential for *vps29* seeds to ensure protein turnover. Indeed, mRNA translational (GO: 0006412) and proteolysis (GO: 0006508) are overrepresented in the *vps29* seed proteome (Figure 3; Table S3). This suggests that the *vps29* mutant is permanently exhausted by the intense activity of protein degradation and synthesis, requiring a huge energy expenditure. By accelerating the consumption rate of reserves that are already reduced (see below), this general high level of energy metabolism in *vps29* seeds may explain the low germination vigor and the shortened longevity of the mutant seeds. 

### 3.3. Cellular Trafficking in vps29 Seeds

In contrast to the embryonic lethality observed in mice harboring a loss of retromer function, *Arabidopsis* retromer mutants can grow, and although exhibiting a severe dwarf phenotype, can generate a few viable seeds [15]. This suggests that other trafficking proteins or pathways can somehow compensate for the lack of retromer function in the *vps29* mutant. Interestingly, the proteomic screening allowed us to identify more than 60 proteins involved in cellular trafficking. Regarding the core retromer components, the lack of VPS29 protein in mutant seeds was associated with a decrease in the amount of VPS26a (At5g53530, ↘2.1) and VPS26b (At4g27690, ↘1.72), whereas VPS35b (At1g75850) was not detected (Tables S1 and S2). The decrease in the core retromer VPS proteins was expected, as we previously showed by immunoblot analysis that VPS35a was less abundant in *vps29* mutants and even undetectable in *vps26a vps26b* double-mutant plants [18]. The sorting nexin1 (SNX1; At5g06140) protein, which can work with the core retromer complex in vacuolar protein trafficking, was as abundant in the Col-0 as in *vps29* seeds, confirming again the results of previous immunoblot analysis [18]. These data indicate that the stoichiometry of the retromer VPS proteins is finely regulated, with the absence of VPS29 or VPS26 proteins leading to a concomitant reduction or lack of the remaining VPS35 proteins, respectively. By contrast, the biosynthesis of SNX1 appears unlinked to that of retromer VPS components. Among the other proteins found in *vps29* and that are implicated in intracellular trafficking, nine were not detected in Col-0 seeds, although we cannot exclude that they were actually present, but only in minute amounts. These include several proteins involved in vesicle-mediated transport (e.g., RABF2b, ARF-GEF, and MIN7) or in the transport of ions and metabolites as well as establishment of protein localization (Figure 3; Table S3). Of the remaining up-accumulated proteins, some are related to the transport or fusion of vesicles, such as VAMP (At3g60600, ↗12), ENTH/ANTH/VHS superfamily protein (At4g32285, ↗7.7), plant VAP homolog 12 (At2g45140, ↗6.0), coatomer α-subunit (At2g21390, ↗7.5; At1g62020, ↗7.5), and the SNARE VTI11 (At5g39510, ↗2.3), or to endocytosis, such as dynamin-like proteins (DRP2B At1g59610, ↗3.6; DRP1E At3g60190, ↗3.1; and DL1C At1g14830, specific to *vps29* seeds).

The up-accumulation of 18 proteins belonging to the RAB GTPase family in *vps29* seeds is particularly noteworthy (Figure 3, Table 3, and Table S2). RAB GTPases, with SNAREs, are the main group of proteins involved in vacuolar trafficking [37]. In *Arabidopsis*, members of the RABF and RABG families are major regulators of the transport of proteins from endosomes to vacuoles, and RABF1 (At3g54840, ↗5.25), RABF2b (At4g19640, only detected in *vps29*), RABG3e (At1g49300, ↗3.22), and RABG3f (At3g18820, ↗3.1) were overrepresented in the mutant. Interestingly, the membrane-associated form of RABG3f was previously shown to physically interact with the retromer subunit VPS35 in plants [18]. 

Altogether, these data suggest that the accumulation of RAB proteins might somehow partially compensate for the loss of retromer function so as to maintain a certain level of transport of compounds from the endosomes to vacuoles. The functional link between RABG3 members and the retromer VPS complex is also supported by the observation that a dominant-negative mutant of RABG3f (RABG3f^T22N^) [39] or a quintuple mutant *rabg3b 3c 3d 3e 3f* [40] exhibit defects both in vacuolar trafficking and the morphology of MVBs and vacuoles, similar to those described in *vps29*, including accumulation of unprocessed precursors of 12S globulin and misrouting of vacuolar proteins to the extracellular space [16,18]. Mazel and collaborators [41] showed that *RABG3e* gene expression was induced by combined treatment with superoxide and salicylic acid or by necrogenic pathogens. Moreover, overexpression of *RABG3e* induces salt and osmotic tolerance in transgenic plants. Together, these results suggest that the three-fold higher abundance of RABG3e in *vps29* might reflect the establishment of a stress response in the mutant seeds. 

VSRs are membrane-anchored proteins that transport cargo proteins from endosomes to the protein storage or lytic vacuoles [42]. We found that loss of VPS29 function was accompanied by an increase in VSR1 (At3g52850, ↗2.36), but not of the two other VSR2 (At2g14720, ↗1.13) and VSR3 (At2g14740, ↗1.19) proteins. Increased abundance of VSR1 in seeds was already reported in *vps29* and *vps35* mutants as measured by immunoblot analysis [43]. Besides, *vsr1* mutants, such as *vps29* and *vps35* mutants, accumulate storage protein precursors and misroute 12S globulin and 2S albumin, which are both secreted to the apoplast [43]. Recycling of VSR1 from the (pre-vacuolar compartment) PVC to the TGN was shown to involve VPS29 [44]. In addition, overexpression of VSR1 in protoplasts derived from a weak *vps29* allele was shown to rescue the defect in vacuolar trafficking. These results, combined with our data, enable us to propose that the increased amount of VSR1 in *vps29* seeds might be a biological response to compensate for retromer inactivation and hence for maintaining vacuolar trafficking through a retromer-independent pathway. Recently, the adaptor protein complex AP-4 was demonstrated to interact with VSR1 and possibly regulate proper localization of VSR1 to the TGN [45]. Null mutations in AP-4 components, such as in AP-4M (At4g24550), lead to VSR1 accumulation and missorting of 12S globulin, but not 2S albumin. We found a strong decrease in AP-4M (At4g24550, ↘ 4.5) abundance in *vps29*, which correlated with VSR1 accumulation and underscores the functional link between the retromer, VSR1, AP-4, and sorting of storage proteins to PSVs. Although *vsr1*, *ap-4*, and retromer *vps* mutants exhibit the accumulation and secretion of precursors of storage proteins, there are still mature forms of these proteins in mutant seeds, indicating that the processing enzymes are properly transported to the vacuoles [8,9,16,18,45]. This implies that the vacuolar processing proteases can be routed independently of VSR1, AP-4, and retromer proteins. Despite the defect observed in the SSP processing (Figure 2), *vps29* seeds showed increased abundance of the beta vacuolar processing enzyme (βVPE; At1g62710, ↗2.45), which is a seed-specific protease involved in the processing of 12S globulin and 2S albumin [46]. Here, again, it is tempting to conclude that this increase counteracts the misrouting of storage proteins in some way. 

Interestingly, we noticed an increased abundance of proteins that mediate the transport of a variety of compounds, ranging from ions to proteins and lipids, some of which have been associated with biotic or abiotic stresses. Among the largest changes in protein abundance were H^+^-ATPase 1 (At2g18960, ↗23.5), proton pump interactor 1 (At4g27500, ↗15), nucleoporin autopeptidase (At1g10390, ↗12), B-cell receptor-associated 31-like (At5g42570, ↗11), vacuolar H^+^-pyrophosphatase 2 (At1g78920, ↗9), multidrug-resistance-associated protein 10 (At3g62700, ↗8), nuclear shuttle protein (NSP)-interacting GTPase (At4g13350, ↗6), and lipid transfer protein 2 (LTP2; At2g38530, ↗5.2). Increase in *LTP2* expression following osmotic stress was previously reported to relate to drought stress response [47]. Finally, the increase in NBR1 (At4g24690, ↗4.33), which is involved in the selective routing of proteins to the vacuole during the autophagy process, is also indicative of a stress situation in the mutant [48].

### 3.4. Storage Processing and Mobilization

The shotgun proteomic approach gives clear indications that the processing of lipid and protein reserves was altered in *vps29* seeds (Figure 3, Table S2). For instance, a large proportion of the changes in the *vps29* seed proteome concerns enzymes involved in lipid metabolism (Figure 3b). Interestingly, our previous work reported that *vps29* mutant seeds are altered in lipid storage and degradation [17]. In addition to a lower fatty acid content and the presence of smaller lipid bodies, we showed that the translocation from peroxisomes to OBs of the main lipase responsible for OB degradation, the SUGAR-DEPENDENT1 (SDP1), is delayed in the *vps29* retromer mutants [17]. The degradation of seed lipid reserve involves the process of β-oxidation that takes place in peroxisomes. In *vps29* seeds, we previously showed that this process is functional, and hence is not responsible for the defects in lipid reserve mobilization observed in *vps29* mutants [17]. Among the four main enzymes involved in β-oxidation, we found modifications in the abundance of enoyl-CoA hydratases (At3g06860, ↗2.1; At4g14430, ↗3.6), acyl-CoA oxidases (At4g16760, ↗3.1; At3g51840, ↘2.5), a 3-hydroxyacyl-CoA dehydrogenase (At3g15290, ↘2.3), and acetyl-CoA carboxylase 1(At1g36160, specific to *vps29* seeds). The amount of the GLABRA2 expression modulator (GEM, At2g22475, ↗7) was much higher in *vps29*. It has been reported that when *GEM* is not expressed in *Arabidopsis*, an 8% increase in the oil content of seeds occurs [49]. Shi et al [50] suggested that this was due to a drift in the carbon allocation from resources normally used for the synthesis of mucilage. Although the link between GEM function and lipid content in seeds remains to be elucidated, the high abundance of GEM in *vps29* might contribute to the altered lipid mobilization observed in the retromer mutant. Similarly, several GDSL-like lipases have a modified abundance in *vps29* seeds: three isoforms of the GDSL-like lipase/acylhydrolase superfamily (At5g03610, At2g27360, and At5g45670) were only detected in the mutant, whereas two GDSL-like lipase/acylhydrolase family proteins were less abundant (At1g29660, ↘3, and At5g03820, ↘2.1). Many functions have been proposed for GDSL family proteins, including stress responses, polysaccharide degradation, and TAG mobilization [51,52,53]. Remarkably, the phosphatidic acid phosphohydrolase 1 (At3g09560, ↗9) was highly up-accumulated in the mutant, which might potentially lead to repression of phospholipid biosynthetic gene expression and hence to changes in membrane lipid homeostasis [54]. 

Confirming the previous work on the effects of retromer dysfunction on seed storage proteins, the seed proteome changes in *vps29* mutant indicate a deeply modified processing of storage proteins (Figure 2). Comparison of the 2D electrophoretic patterns of seed proteomes with the reference database shows a decrease in abundance of 12S α- and β-cruciferin spots in *vps29* seeds (Figure 2). Concomitantly, several spots corresponding to precursors of these storage proteins, such as 12S seed storage protein precursors, were more abundant in *vps29* seeds, indicating that defects in retromer function do not alter seed storage protein production, but instead inhibit their maturation. Indeed, the shotgun proteome analysis showed a similar quantity of peptides belonging to 12S cruciferins in *vps29* and Col-0 seeds (Table S1), which is consistent with an increased abundance of precursors, but an inhibition of their maturation, as already reported for *vps29* [9]. Contrariwise, a rise in the amount of several seed storage albumins was observed (At4g27140, ↗4.4; At5g54740, ↗3.5; At4g27170, ↗2.7; At4g27160, ↗2.2; At4g27150, ↗2.2; Table S2), highlighting that *vps29* mutation affects storage protein accumulation in different ways.

Interestingly, the proteomic analysis also reveals that several proteins involved in protein processing and/or degradation had increased abundance in *vps29*. Indeed, cysteine proteases and their precursors (At4g11320, ↗11.4; At3g54940, ↗4.1; At4g11310, ↗9; At4g01610, only detected in *vps29*) and proteins related to ubiquitination (At1g70320, ↗5.3; At5g37640, ↗3.1; At5g42220, ↗3; At1g31340, ↗2.8; At3g20630, ↗2) and/or to proteasome activity (At2g20580, ↗2.1; At2g47940, ↗3; At3g13330 only in *vps29* seeds) were significantly more abundant in *vps29* seeds. These data suggest that the retromer not only functions in protein trafficking and maturation, but is likely to mediate protein turnover through ubiquitination- and proteasome-dependent pathways.

### 3.5. Changes in Cell Wall Metabolism

The strongest modification found in the proteomic comparison between *vps29* and Col-0 seeds was for β-glucosidase 25 (At3g03640), which was over 40 times (↗41.3) more abundant in the mutant seeds. However, the lack of the conserved Glu residue essential for hydrolase activity questions its actual function as a glucosidase (https://www.uniprot.org/uniprot/O82772-1). The content of other β-glucosidases was also affected in *vps29*, showing either increased (At4g22100, ↗6; At1g02850, ↗3; At3g18080, ↗2.1) or decreased (At5g25980, ↘5.3) levels. These enzymes catalyze the degradation of cellulose or xyloglucans [55], which both consist of polyoside units of anhydroglucose. The increased abundance of anhydroglucose (↗5.8; Table 2) further suggests that hydrolysis of these polymers does occur in *vps29* seeds. Some glycosyl hydrolase family proteins also had increased levels, with three isoforms of β-glucanases detected only in the mutant seeds (At5g42720, At3g55430, and At2g05790) and a forth isoform being slightly more abundant (At5g04885, ↗2.1). This family of proteins may have a role in the metabolism of xyloglucans [56]. This may also be the case for expansin-like A1 (At3g45970, ↗5.5), because its putative substrate is the cellulose–xyloglucan link [57]. Finally, one xyloglucan endotransglucosylase/hydrolase 11 (At3g48580, ↗2.6) plays a role in cellulose deposition in the cell wall [58]. In dicots, xyloglucans are the major form of hemicellulose in the cell wall, contributing up to 20% of the dry weight. Noncovalently bound to cellulose, and related to cell expansion, the xyloglucans affect the ability of the cell to grow under osmotic constraints.

The content in rhamnose was reduced in *vps29* seeds (↘2.7) in spite of a greater abundance of a rhamnose biosynthesis 1 (At1g78570, ↗6.5) and a UDP-4-keto-6-deoxy-d-glucose-3,5-epimerase-4-reductase 1 (At1g63000, ↗7). This suggests that rhamnose was transformed, maybe through the pectin synthesis pathway. Indeed, the proteome of *vps29* seeds showed increased abundance of proteins involved in pectin metabolism, which is a major component of the cell wall. Among the strongest modifications are proteins having the domain of unknown function DUF642 (At1g29980, ↗15; At3g08030, ↗2.9; At5g25460, ↘21; At5g11420, ↘20). The DUF642 family proteins are reported to localize in the cell wall polysaccharide fraction [59], where At5g11420 interacts in vitro with the catalytic domain of the pectin methylesterase AtPME3 [60]. Surprisingly, although *At3g08030* transcripts have been described as a marker for seed germination performance [61], *vps29* seeds, which have higher levels of the protein compared with the wild-type, exhibit a lower vigor. We may hypothesize that the other strong alterations observed in the proteome of the mutant seeds prevent the beneficial action of *At3g08030* in germination.

Other enzymes involved in the synthesis of cell wall components were also increased in abundance in the mutant seeds. For example, the glucuronokinase (At3g01640, ↗3.8), in association with a pyrophosphorylase (At5g09650, ↗2.2), catalyzes the transformation of myo-inositol (↗3.4) into glucuronate (not quantified). Then, the UDP–glucuronic acid decarboxylase 2 (At3g62830, ↗7.5) produces xylose (↗2), which in turn enters the composition of pectin. The glycerophosphodiester phosphodiesterase (At4g26690 ↗10), which is strongly increased in abundance in *vps29*, is involved in the crosslinking of pectic polysaccharides [62]. Other modifications concern proteins of the pectin acetylesterase family (At5g45280, ↗2.2; At4g19410, ↗2.2) and of the plant invertase/pectin methylesterase inhibitor (PMEI) superfamily (At3g17130, ↗5.5; At3g60730, ↗3). PMEIs are also reported to be responsive to osmotic stress [63]. Ribophorin I (At2g01720, ↗10) and Hapless 6 (At4g21150, ↗7.8), which contains a ribophorin II domain, have a putative role in the synthesis of pectin [64]. In the proteome of *vps29* seeds, some subtilases were more abundant (At5g67360, ↗5.5; At2g05920, ↗2.7; At3g14240, only detected in the mutant) or less abundant (At4g21650, only detected in Col-0 seeds). Subtilases are serine proteases involved in cell wall structure and mucilage synthesis just before germination. Interestingly, *Arabidopsis* mutant seeds lacking the functional subtilase AtSBT1.7 (At5g67360) have an altered mode of rupture of the outer seed coat wall associated with an increased pectin methylesterase activity [65]. This latter observation indicates a role for subtilases and PME/PMEI in the loosening of the seed outer primary cell wall. Rearrangements of cell wall metabolism observed in *vps29* seeds may affect cell expansion, leading to radicle protrusion and thus reducing germination success.

## 4. Materials and Methods

### 4.1. Plant Material and Culture Conditions

The *Arabidopsis thaliana* Columbia 0 (Col-0) accession was used as the wild-type (WT). *vps29-4* mutant seeds were obtained from Prof. Dr. Bernd Weisshaar (MPI for Plant Breeding Research, Cologne, Germany) and have been described previously [15]. For seed production, plants were grown in a growth chamber at 19/21 °C under a 16-h photoperiod of artificial light (Orsam L58/31830 luminux plus Wanton Wan White tubes, 45 μmol m^−2^ s^−1^) and 70% relative humidity (RH). After complete seed maturation, plants were no longer watered and were kept dry for 3 weeks before seed harvest. The collected dry seeds were then stored for 2 months at 4 °C, 50% (Relative Humidity) RH, and in darkness prior to any experiment.

### 4.2. Germination Assays

Germination assays were carried out on three replicates (independent experiments) of 100 seeds on a basal medium composed of distilled water buffered with MES (3 mM 4-morpholineethanesulfonic acid, pH 5.7) and gellified with agar (7 g/L agar HP 696; Kalys Biotech.) in covered plastic boxes (Ø 50 mm). Seeds were placed in cold stratification conditions for 4 days at 4 °C in darkness and then transferred to a controlled culture cabinet under continuous light (Philips TRM HOW/33RS tubes, 170 μmol m^−2^ s^−1^), at 25 °C, and a constant 70% RH. Germination (considered as radicle protrusion through the seed coat and the endosperm) was scored daily. To address seed longevity, a controlled deterioration treatment (CDT) was applied as described by Rajjou et al. [66].

### 4.3. Total Soluble Protein Extraction

Total protein extracts were obtained from dry mature seeds as described by Rajjou et al. [21]. Fifty milligrams of dry mature seeds was ground with a mortar and pestle to a fine powder in liquid nitrogen. Proteins were extracted with 320 µL of extraction buffer (18 mM Tris–HCl, 14 mM Tris–Base, 0.2% (*v*/*v*) Triton X-100, 7 M urea, 2 M thiourea, 60 mM CHAPS), to which was added 53.5 µL of a protease inhibitor cocktail (Complete Mini EDTA-free, Roche Diagnostics GmbH, Pleasanton, CA, USA), 7.5 µL of 1 M DTT, 2 µL (20 units) of DNAse I (Sigma, St. Louis, MO, USA), and 12 µL (2000 units) of RNAse A (Sigma). After gentle agitation for 1 h at 4 °C, samples were centrifuged (20,000× *g*, 15 min). The supernatant was centrifuged the same way a second time. A Bradford protein assay [67] was used to measure protein concentration of the extracts with bovine serum albumin as a standard.

### 4.4. 2D Gel-Based Proteomics and Image Processing

For each genotype, 2D gels were made in two technical replicates from two independent protein extractions (two biological replicates). The gel strips (Immobiline Dry Strip pH 3–10 NL, 24 cm, GE Healthcare Life Sciences, Uppsala, Sweden) were rehydrated for 15 h at room temperature with rehydration buffer (18 mM Tris–HCl, 14 mM Tris–Base, 2.2% (*v*/*v*) Triton X-100, 7 M urea, 2 M thiourea, 60 mM CHAPS, 20 mM DTT, 1% (*v*/*v*) Pharmalyte pH 3–10) added with 150 µg of protein extract to obtain a final volume of 450 µL. Isoelectrofocusing was performed using IPGphore (GE Healthcare) following the program: 4 h at 60 V, 4 h at 500 V, 2 h of gradient increase to 1000 V, 3.5 h of gradient increase to 10,000 V, then constant voltage at 10,000 V until reaching 92 kV·h^−1^. Strips were stored at −20 °C. They were equilibrated first for 30 min in the equilibration solution composed of 6 M urea, 150 mM Bis–Tris, 0.1 M HCl pH 8.8, 30% (*v*/*v*) glycerol, and 2.5% (*w*/*v*) SDS added with 50 mM DTT. They were then placed for 5 min in equilibration solution, followed by a final bath in the same solution with 4% iodoacetamide (*w*/*v*) added for 20 min. The equilibrated strips were loaded on acrylamide gels with a 1% (*w*/*v*) agarose, 150 mM Bis–Tris, and 0.4% (*w*/*v*) SDS solution and the SDS-PAGE was performed on 10% polyacrylamide gels containing 10% (*v*/*v*) acrylamide, 0.33% (*w*/*v*) diacrolyl piperazine, 0.18 M Tris, 0.166 M HCl pH 8.3, 0.07% (*w*/*v*) ammonium persulfate, 0.035% (*v*/*v*) tetramethylethylenediamine, and 2% glycerol in a running buffer composed of 25 mM Trizma-Base, 200 mM taurine, 0.15% (*w*/*v*) thiosulfate, and 0.1% (*w*/*v*) SDS. After protein separation, the gels were silver-stained according to [21]. The stained gels were scanned using an Epson Perfection V700 scanner at 300 dpi. Quantitative analysis of the 2D gels was then achieved using the Progenesis SameSpot software (v3.2, NonLinear Dynamics, UK) as previously described [68]. The 2D reference maps available on the *Arabidopsis* seed proteome web portal (www.seed-proteome.com; [22]) were used for matching with the spots of interest, with a particular attention to the easily detectable SSP isoforms.

### 4.5. Shotgun Proteomic Analysis

The proteome exploration was enriched by a quantitative LC–MS/MS analysis. Thirty micrograms of soluble protein extracts (n = 3 biological replicates) were subjected to 1D SDS–PAGE. Protein extracts were loaded in 1 × Laemmli buffer [69] with DTT (50 mM) in a 12% acrylamide gel (563 mM Tris–HCl, pH 8.8, 0.1% (*w*/*v*) SDS). After 90 min of migration at 10 mA, the gel was stained with colloidal blue (GelCode Blue Stain Reagent; Thermo Fisher Scientific Inc, Rockford, IL, USA) and destained in MilliQ water. Each lane was systematically cut into 14 bands with a grid cutter. Thus, 84 (6 × 14) gel bands were prepared for LC–MS/MS analysis. Each gel band was submitted to in-gel digestion with the Progest system (Genomic Solution, Huntingdon, UK), according to a standard trypsin protocol. Briefly, gel pieces were washed with 25% (*v*/*v*) acetonitrile, 50 mM ammonium bicarbonate, pH 7.8 for 60 min at 37 °C, followed by dehydration with 100% acetonitrile for 15 min. Gel pieces were rehydrated overnight at 37 °C with 1/50 (*w*/*w*) trypsin (Promega, Madison, WI, USA) in 20 mM ammonium bicarbonate, pH 7.8. Digestion was stopped by adding 0.4% (*v*/*v*) of trifluoroacetic acid (TFA). HPLC was performed on a NanoLC-Ultra system (Eksigent, Dublin, CA, USA). A 4-µL sample was loaded at 7.5 µL/min on a precolumn cartridge (stationary phase: BIOSPHERE C18, 5 µm; column: 100 µm i.d., 2 cm; NanoSeparations, Nieuwkoop, NL, USA) and desalted with 0.1% HCOOH. After 3 min, the precolumn cartridge was connected to the separating PepMap C18 column (stationary phase: BIOSPHERE C18, 3 µm; column: 75 µm i.d., 150 mm; NanoSeparations). Buffers were 0.1% HCOOH in water (A) and 0.1% HCOOH in ACN (B). The peptide separation was achieved with a linear gradient from 5 to 30% B for 30 min at 300 nL/min. Including the regeneration step at 95% B and the equilibration step at 95% A, one run took 45 min. Eluted peptides were analyzed online with a Q-Exactive mass spectrometer (Thermo Electron, Bremen, Germany) using a nanoelectrospray interface (noncoated capillary probe, 10 µm i.d.; New Objective, Cambridge, MA, USA). The Xcalibur 2.1 interface was used to monitor data-dependent acquisition of peptide ions. This included a full MS scan covering 300 to 1400 range of mass-to-charge ratio (*m*/*z*) with a resolution of 70,000 and a tandem mass spectrometry (MS/MS) step (normalized collision energy: 30%; resolution: 17,500). The MS/MS step was reiterated for the 8 major ions detected during the full MS scan. Dynamic exclusion was set to 45 s.

### 4.6. Protein Identification and Quantification

A database search was performed with XTandem (http://www.thegpm.org/TANDEM/). Enzymatic cleavage was declared as a trypsin digestion with two possible missed cleavages. Carbamidomethylation of cysteine residues was set to static modifications. Methionine oxidation was set as a possible modification. Precursor mass and fragment mass tolerance were 10 ppm and 0.02 Da, respectively. A refinement search was added with similar parameters, except that semitrypsic peptides and possible N-terminal protein acetylation or deamidation were searched for. The *Arabidopsis* genome database (http://www.arabidopsis.org) and a contaminant database (trypsin, keratins) were used. Only peptides with an E value smaller than 0.05 were reported. Identified proteins were analyzed using XTandem (http://www.thegpm.org). Changes at the protein abundance level between Col-0 and *vps29* seeds were further monitored by spectral counting. The protein abundance index (PAI), as the number of observed peptides divided by the number of observable peptides per protein, was used to estimate the absolute protein contents [70,71]. Proteins were regarded as differentially accumulated in the mutant when their relative abundance changed by a ratio of at least 2 and when they had a *p*-value (nonparametric Kruskal–Wallis k-samples test) less than or equal to 0.05. 

### 4.7. Metabolome Analysis by Gas Chromatography Coupled to Mass Spectrometry (GC–MS)

Metabolite samples were obtained starting from three replicates of 20 mg of *Arabidopsis* dry mature seeds. Seeds were ground with mortar and pestle in liquid nitrogen and stored at −80 °C. Each sample was placed in 2 mL Safelock Eppendorf tubes (Eppendorf AG, Hamburg, Germany). The ground frozen samples were resuspended in 1 mL of water/acetonitrile/isopropanol (2:3:3) (−20 °C) containing ribitol at 4 µg/mL and extracted for 10 min at 4 °C with shaking at 1400 rpm in an Eppendorf Thermomixer (Eppendorf). Insoluble material was removed by centrifugation at 20,000× *g* for 5 min. Twenty-five microliters were collected and dried for 150 min in a SpeedVac (Savant SPD131DDA, Thermo Fisher Scientific, Waltham, MA, USA) and stored at −80 °C. Samples were taken out of −80 °C storage, warmed 15 min before opening, and speed-vac dried for 1 h before adding 10 µL of 20 mg/mL methoxyamine in pyridine to the samples, the blanks, and amino acid and sugar standards. The reaction was performed for 90 min at 28 °C under continuous shaking in an Eppendorf thermomixer. Ninety microliters of N-methyl–N-trimethylsilyl–trifluoroacetamide (MSTFA) were then added and the reaction continued for 30 min at 37 °C. After cooling, 50 µL were transferred to an Agilent vial for injection. Two hours after derivatization, 1 µL of sample was injected in splitless mode into an Agilent 7890A gas chromatograph coupled to an Agilent 5975C mass spectrometer. The column was an Rxi-5SilMS from Restek (30 m with 10 m integra-guard column). The liner (Restek # 20994) was changed before each series of analysis, and 10 cm of column were cut. Oven temperature ramp was 70 °C for 7 min, and then increased by 10 °C/min to 325 °C, which was held for 4 min (run length: 36.5 min). Helium constant flow was 1.52 mL/min. Temperatures were the following: injector: 250 °C, transfer line: 290 °C, source: 250 °C, and quadripole: 150 °C. Samples and blanks were randomized. Amino acid standards were injected at the beginning and end of the analysis for monitoring of the derivatization stability. An alkane mix (C10, C12, C15, C19, C22, C28, C32, and C36) was injected into the middle of the queue for external (Retention index) RI calibration. Five scans per second were acquired. Raw Agilent datafiles were converted into NetCDF format and analyzed with AMDIS (http://chemdata.nist.gov/mass-spc/amdis/). A home retention indices/mass spectra library was used for metabolite identification. Peak areas were then determined using the QuanLynx software (Waters Corporation, Milfords, MA, USA) after conversion of the NetCDF file into MassLynx (Waters) format. Two analytical replicates were made for each biological replicates; we thus made the statistical analysis on 6 samples of the WT and 6 samples of *vps29*. A Kruskal–Wallis test and a Benjamini and Hochberg false discovery rate (FDR) adjustment were used with a significance level set at 5%.

## 5. Conclusions

**Figure 5:**
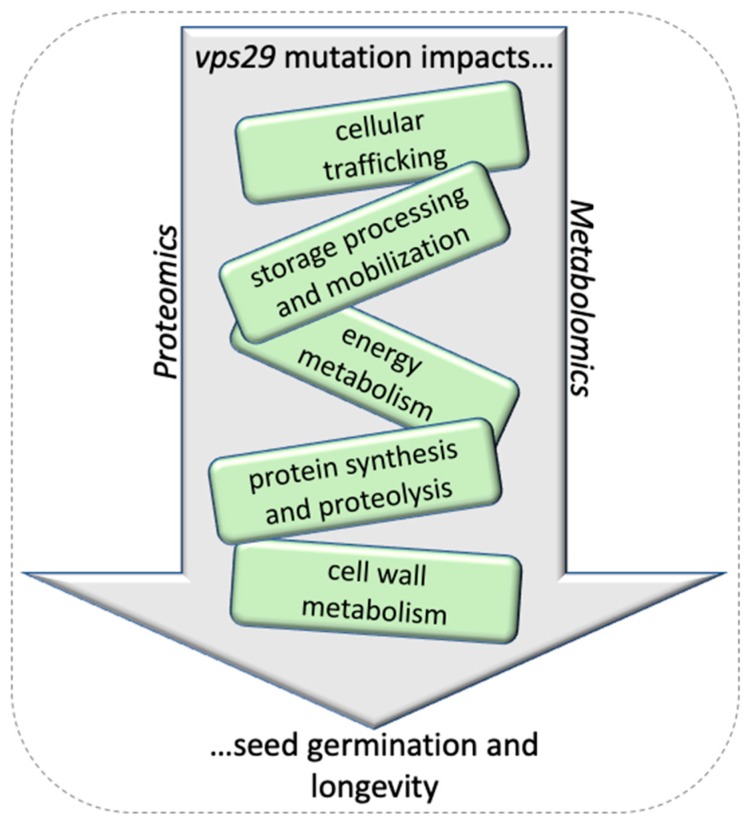
Schematic diagram illustrating the main effects related to *vps29* mutation in *Arabidopsis thaliana* seeds highlighted by proteomics and metabolomics.

The present study focuses on the seed stage, which is the final step of the life cycle of a flowering plant and integrates all the events affecting the *vps29* mutant following fertilization and seed development. Our comparative analysis of the Col-0 wild-type and *vps29* mutant seeds, by combining information from the study of the phenotype, proteome, and metabolome, provides a massive and coherent set of data that reveals a considerable impact of the *vps29* mutation on cell function (Figure 5). Taken together, the major changes observed in comparing the metabolome and proteome of wild-type versus *vps29* seeds indicate that a stress response is ongoing in the seeds of *vps29* plants. Given the large range of alterations in protein trafficking associated with the loss of VPS29/retromer function, it is likely that this strongly impairs communication inside and between cells, leading to the severe developmental defects reported [15] and the emergence of a global stress response as described here. The establishment of oxidative stress may have been facilitated by the low content of processed storage proteins, and in particular of the 12S cruciferin α- and β-subunits, which act as a buffer against biomolecule damage resulting from the oxidative stress occurring during dry storage and germination [72,73].

The loss of a functional retromer complex leads to a deep modification of cellular trafficking, which seriously affects lipid and protein storage as well as several biosynthetic pathways, including those involved in cell wall composition and structure. Because of the significant increase in xyloglucan-degradative enzymes found in *vps29* seeds, a decrease in the amount of cell wall xyloglucans is likely to occur. This probably induces decreased stiffness of the cell wall, as reported in several cell wall mutants [74], which could be the source of a stress signaling pathway [75]. Based on our results, we make the hypothesis that in *vps29* seeds, alteration of the cell wall, associated with decreased abundance of oxidative-protectant storage proteins and an increased level of energy metabolism, might at least partly account for the implementation of an osmotic stress in the retromer mutant seeds. Oxidative stress ordinarily takes part in the signaling pathway that induces germination [76]. In *vps29* seeds, we found less α-tocopherol and proline than usual, which are markers of the dry seed stage, as well as a high energy metabolism and content in amino acids, which are traits of ongoing germination. Protein turnover appears highly enhanced in the *vps29* mutant, requiring high energy costs. Together with the impaired protein maturation necessary for proper germination, this could explain both the delay in germination and the reduced time span of seed viability observed in *vps29* seeds. Our study reveals that combining seed proteomics and metabolomics provides invaluable information to better characterize mutant plants, which was not predicted from the phenotypic analysis, and opens new directions of research to decipher how the function of a particular protein is integrated in the global functional network of an organism. 

## Figures and Tables

**Table 1:** Metabolites significantly up-accumulated in Col-0 dry seeds by comparison with *vps29* dry seeds. A Kruskal–Wallis test and a Benjamini and Hochberg false discovery rate (FDR) adjustment were used with a significance level set at 5%. Significant *p*-values are indicated in bold. RI, retention index obtained from GC/MS analysis. Metabolites that were detected, but with no significant change in abundance between genotypes, are presented in the Supplementary Materials (Table S4).

Metabolites	Abundance in Col-0 Seeds (Mean)	Abundance in *vps29* Seeds (Mean)	Fold Change (Col-o/*vps29*)	*p*-Value	FDR-Adjusted *p*-Value
β-Alanine	4.84	5.35 × 10^−1^	9.1	0.002	0.007
Hexacosanoate	2.14 × 10^−4^	3.19 × 10^−5^	6.7	0.009	0.022
β-Aminoisobutyrate	6.39 × 10^−2^	1.14 × 10^−2^	5.6	0.002	0.007
Dibenzoyltartrateanhydre	3.06 × 10^−1^	8.48 × 10^−2^	3.6	0.003	0.007
Monosearin	6.63 × 10^−4^	1.89 × 10^−4^	3.5	0.003	0.007
Glutamine	1.00	3.25 × 10^−1^	3.1	0.002	0.007
Maleate	1.12 × 10^−3^	3.73 × 10^−4^	3.0	0.002	0.007
Rhamnose	2.10 × 10^−1^	7.75 × 10^−2^	2.7	0.002	0.007
2-Oxoglutarate	3.37 × 10^−2^	1.24 × 10^−2^	2.7	0.002	0.007
Alpha-tocopherol	1.13 × 10^−2^	4.29 × 10^−3^	2.6	0.002	0.007
Malonate	1.38 × 10^−1^	5.41 × 10^−2^	2.6	0.040	0.079
Phenylalanine	3.29 × 10^−1^	1.31 × 10^−1^	2.5	0.002	0.007
Cystine	1.65 × 10^−3^	7.07 × 10^−4^	2.3	0.002	0.007
β-Indole-3-acetonitrile	2.90 × 10^−2^	1.31 × 10^−2^	2.2	0.002	0.007
Fumarate	1.34	6.40 × 10^−1^	2.1	0.002	0.007
Unknown sugar (RI = 2550.4; mass = 219)	2.18 × 10^−3^	1.19 × 10^−3^	1.8	0.002	0.007
Proline	2.12	1.18	1.8	0.004	0.011
Urea	7.82 × 10^−3^	4.47 × 10^−3^	1.7	0.002	0.007
Malate	2.35	1.38	1.7	0.002	0.007
Nicotinate	7.61 × 10^−3^	4.98 × 10^−3^	1.5	0.002	0.007
Glutamate	2.11	1.45	1.5	0.009	0.022
Citrate	4.42 × 10^−1^	3.28 × 10^−1^	1.3	0.002	0.007
Dibenzoyltartrate	8.48 × 10^−2^	6.34 × 10^−2^	1.3	0.002	0.007
Kaempferol	5.52 × 10^−4^	4.45 × 10^−4^	1.2	0.026	0.056

**Table 2:** Metabolites significantly up-accumulated in *vps29* dry seeds by comparison with Col-0 dry seeds. A Kruskal–Wallis test and a Benjamini and Hochberg false discovery rate (FDR) adjustment were used with a significance level set at 5%. Significant *p*-values are indicated in bold. RI, retention index obtained from GC/MS analysis. Metabolites that were detected but with no significant change in abundance between genotypes are presented in the Supplementary Materials (Table S4).

Metabolites	Abundance in Col-0 Seeds (Mean)	Abundance in *vps29* Seeds (Mean)	Fold Change (Col-o/*vps29*)	*p*-Value	FDR-Adjusted *p*-Value
Xylitol	4.14 × 10^−3^	1.08 × 10^−1^	26.15	0.002	0.007
Sorbitol	6.43 × 10^−2^	1.42	22.11	0.002	0.007
Trehalose	7.43 × 10^−4^	1.44 × 10^−2^	19.39	0.002	0.007
Epicatechin	2.33 × 10^−3^	3.50 × 10^−2^	15.04	0.002	0.007
Putrescine	6.39 × 10^−3^	5.62 × 10^−2^	8.79	0.002	0.007
Alanine	4.93 × 10^−1^	3.21	6.51	0.002	0.007
Oleic acid	1.18 × 10^−3^	7.40 × 10^−3^	6.25	0.002	0.007
Anhydroglucose	1.99 × 10^−2^	1.15 × 10^−1^	5.81	0.002	0.007
Lysine	8.74 × 10^−3^	4.93 × 10^−2^	5.64	0.002	0.007
Gluconate	9.07 × 10^−1^	5.07	5.59	0.002	0.007
Gamma-hydroxybutyric acid	1.81 × 10^−4^	9.00 × 10^−4^	4.96	0.002	0.007
Pipecolate	3.55 × 10^−3^	1.69 × 10^−2^	4.76	0.002	0.007
Linoleic acid	2.34 × 10^−3^	9.16 × 10^−3^	3.91	0.002	0.007
Glycine	1.51 × 10^−1^	5.63 × 10^−1^	3.73	0.002	0.007
Myo-inositol	3.54 × 10^−1^	1.21	3.41	0.002	0.007
Succinate	1.85 × 10^−1^	6.00 × 10^−1^	3.24	0.002	0.007
Glycerate	4.70 × 10^−2^	1.48 × 10^−1^	3.14	0.002	0.007
Glycerol	2.62 × 10^−2^	7.81 × 10^−2^	2.98	0.002	0.007
Linolenic acid	6.01 × 10^−4^	1.77 × 10^−3^	2.95	0.002	0.007
Serine	2.41 × 10^−1^	7.08 × 10^−1^	2.94	0.002	0.007
Glycerol-3-phosphate	1.43 × 10^−2^	3.95 × 10^−2^	2.76	0.002	0.007
Arabitol	4.38 × 10^−2^	1.11 × 10^−1^	2.53	0.002	0.007
7-(Methylthio)heptyl-glucosinolate	1.78 × 10^−3^	4.29 × 10^−3^	2.41	0.039	0.079
Threitol	5.48 × 10^−4^	1.31 × 10^−3^	2.39	0.002	0.007
Tyrosine	2.56 × 10^−2^	5.97 × 10^−2^	2.33	0.002	0.007
Homoserine	2.44 × 10^−3^	5.55 × 10^−3^	2.27	0.002	0.007
8-(Methylthio)octyl-glucosinolate	2.05 × 10^−3^	4.57 × 10^−3^	2.23	0.042	0.079
6-(Methylthio)hexanenitrile	9.16 × 10^−3^	1.90 × 10^−2^	2.07	0.002	0.007
Xylose	5.31 × 10^−2^	1.04 × 10^−1^	1.96	0.002	0.007
Unknown sugar (RI = 3891.7; Mass = 204)	6.17 × 10^−2^	1.20 × 10^−1^	1.95	0.025	0.056
Methionine	4.39 × 10^−2^	8.49 × 10^−2^	1.93	0.002	0.007
8-(Methylthio)octanenitrile	1.51 × 10^−2^	2.88 × 10^−2^	1.90	0.002	0.007
Methionine	4.49 × 10^−3^	8.25 × 10^−3^	1.84	0.026	0.056
Mannose	8.82 × 10^−2^	1.61 × 10^−1^	1.82	0.039	0.079
Arginine	2.86 × 10^−1^	5.15 × 10^−1^	1.80	0.002	0.007
Galactonate	5.81 × 10^−2^	1.01 × 10^−1^	1.75	0.008	0.020
(Gamma-aminobutyric acid) GABA	2.12 × 10^−1^	3.42 × 10^−1^	1.62	0.004	0.011
9-(Methylthio)nonanenitrile	2.08 × 10^−2^	3.36 × 10^−2^	1.61	0.002	0.007
Quercitrin	2.67 × 10^−2^	4.24 × 10^−2^	1.58	0.026	0.056
Valine	5.31 × 10^−1^	8.07 × 10^−1^	1.52	0.002	0.007
Eicosanoate	3.60 × 10^−3^	5.45 × 10^−3^	1.51	0.041	0.079
Allantoin	2.07 × 10^−2^	3.12 × 10^−2^	1.51	0.004	0.011
Galactinol	5.63 × 10^−1^	8.38 × 10^−1^	1.49	0.027	0.056
Sucrose	3.36 × 10^1^	4.96 × 10^1^	1.48	0.029	0.061
Alpha-aminoadipate	4.38 × 10^−2^	6.38 × 10^−2^	1.46	0.026	0.056
Threonate	7.57 × 10^−3^	1.10 × 10^−2^	1.45	0.027	0.056
Ribose	6.02 × 10^−2^	8.62 × 10^−2^	1.43	0.009	0.022
Allantoin	4.78 × 10^−2^	6.58 × 10^−2^	1.38	0.027	0.056
Leucine	1.88 × 10^−1^	2.57 × 10^−1^	1.36	0.002	0.007
Isoleucine	2.32 × 10^−1^	2.98 × 10^−1^	1.28	0.008	0.021
Cis-sinapinate	1.03 × 10^−2^	1.28 × 10^−2^	1.25	0.041	0.079

**Table 3:** Proteins of the RAB GTPase family found to be more abundant in *vps29* seeds.

RAB Name ^a^	AGI Gene ^b^	RAB Abundance Ratio *vps29*/Col-0
**AtRABA1a**	*At1g06400*	2.3
**AtRABA1b**	*At1g16920*	2.5
**AtRABA1c**	*At5g45750*	2.3
**AtRABA2a**	*At1g09630*	2.1
**AtRABA2c**	*At3g46830*	2.5
**AtRABA4a**	*At5g65270*	3.6
**AtRABB1b**	*At4g35860*	3.3
**AtRABB1c**	*At4g17170*	2.1
**AtRABD1**	*At3g11730*	2.0
**AtRABD2a**	*At1g02130*	2.4
**AtRABD2b**	*At5g47200*	3.7
**AtRABD2c**	*At4g17530*	3.2
**AtRABF1**	*At3g54840*	5.3
**AtRABF2b**	*At4g19640*	only detected in *vps29* seeds
**AtRABG3e**	*At1g49300*	3.2
**AtRABG3f**	*At3g18820*	3.1
**AtRABH1b**	*At2g44610*	5.0
**AtRABH1c**	*At4g39890*	4.3

^a^ Nomenclature used by Pereira-Leal and Seabra [38]; ^b^ AGI gene nomenclature.

## Supplementary Materials

Supplementary materials can be found at http://www.mdpi.com/1422-0067/20/2/362/s1.

## Abbreviations

MVB	multivesicular body
PVC	prevacuolar compartment
PSV	protein storage vacuole
SSP	seed storage protein
TGN	trans-Golgi network
VSR	vacuolar sorting receptor

## References

[1] Baud S., Dubreucq B., Miquel M., Rochat C., Lepiniec L. (2008). Storage reserve accumulation in Arabidopsis: Metabolic and developmental control of seed filling. Arabidopsis Book.

[2] Baud S., Boutin J.-P., Miquel M., Lepiniec L., Rochat C. (2002). An integrated overview of seed development in *Arabidopsis thaliana* ecotype WS. Plant Physiol. Biochem..

[3] Baud S., Kelemen Z., Thévenin J., Boulard C., Blanchet S., To A., Payre M., Berger N., Effroy-Cuzzi D., Franco-Zorrilla J.M. (2016). Deciphering the molecular mechanisms underpinning the transcriptional control of gene expression by L-AFL proteins in Arabidopsis seed. Plant Physiol..

[4] Fatihi A., Boulard C., Bouyer D., Baud S., Dubreucq B., Lepiniec L. (2016). Deciphering and modifying LAFL transcriptional regulatory network in seed for improving yield and quality of storage compounds. Plant Sci..

[5] North H., Baud S., Debeaujon I., Dubos C., Dubreucq B., Grappin P., Jullien M., Lepiniec L., Marion-Poll A., Miquel M. (2010). Arabidopsis seed secrets unravelled after a decade of genetic and omics-driven research. Plant J..

[6] Robinson D.G., Hinz G. (1999). Golgi-mediated transport of seed storage proteins. Seed Sci. Res..

[7] Otegui M.S., Spitzer C. (2008). Endosomal functions in plants. Traffic.

[8] Shimada T., Fuji K., Tamura K., Kondo M., Nishimura M., Hara-Nishimura I. (2003). Vacuolar sorting receptor for seed storage proteins in *Arabidopsis thaliana*. Proc. Natl. Acad. Sci. USA.

[9] Shimada T., Koumoto Y., Li L., Yamazaki M., Kondo M., Nishimura M., Hara-Nishimura I. (2006). AtVPS29, a putative component of a retromer complex, is required for the efficient sorting of seed storage proteins. Plant Cell Physiol..

[10] Attar N., Cullen P.J. (2010). The retromer complex. Adv. Enzyme Regul..

[11] McNally K.E., Cullen P.J. (2018). Endosomal retrieval of cargo: Retromer is not alone. Trends Cell Biol..

[12] Seaman M.N.J., Marcusson E.G., Cereghino J.L., Emr S.D. (1997). Endosome to Golgi retrieval of the vacuolar protein sorting receptor, Vps10p, requires the function of the *VPS29*, *VPS30* and *VPS35* gene products. J. Cell Biol..

[13] Seaman M.N.J. (2012). The retromer complex—Endosomal protein recycling and beyond. J. Cell Sci..

[14] Burd C., Cullen P.J. (2014). Retromer: A master conductor of endosome sorting. Cold Spring Harb. Perspect. Biol..

[15] Jaillais Y., Santambrogio M., Rozier F., Fobis-Loisy I., Miège C., Gaude T. (2007). The retromer protein VPS29 links cell polarity and organ initiation in plants. Cell.

[16] Pourcher M., Santambrogio M., Thazar N., Thierry A.-M., Fobis-Loisy I., Miège C., Jaillais Y., Gaude T. (2010). Analyses of SORTING NEXINs reveal distinct retromer-subcomplex functions in development and protein sorting in *Arabidopsis thaliana*. Plant Cell.

[17] Thazar-Poulot N., Miquel M., Fobis-Loisy I., Gaude T. (2015). Peroxisome extensions deliver the *Arabidopsis* SDP1 lipase to oil bodies. Proc. Natl. Acad. Sci. USA.

[18] Zelazny E., Santambrogio M., Pourcher M., Chambrier P., Berne-Dedieu A., Fobis-Loisy I., Miège C., Jaillais Y., Gaude T. (2013). Mechanisms governing the endosomal membrane recruitment of the core retromer in Arabidopsis. Biol. Chem..

[19] Rajjou L., Duval M., Gallardo K., Catusse J., Bally J., Job C., Job D. (2012). Seed germination and vigor. Annu. Rev. Plant Biol..

[20] Finch-Savage W.E., Bassel G.W. (2016). Seed vigour and crop establishment: extending performance beyond adaptation. J. Exp. Bot..

[21] Rajjou L., Belghazi M., Catusse J., Ogé L., Arc E., Godin B., Chibani K., Ali-Rachidi S., Collet B., Grappin P., Kermode A.R. (2011). Proteomics and Posttranslational Proteomics of Seed Dormancy and Germination. Seed Dormancy.

[22] Galland M., Job D., Rajjou L. (2012). The seed proteome web portal. Front Plant Sci..

[23] Galland M., Huguet R., Arc E., Cueff G., Job D., Rajjou L. (2014). Dynamic proteomics emphasizes the importance of selective mRNA translation and protein turnover during Arabidopsis seed germination. Mol. Cell Proteom..

[24] Wang W.Q., Liu S.J., Song S.Q., Møller I.M. (2015). Proteomics of seed development, desiccation tolerance, germination and vigor. Plant Physiol. Biochem..

[25] Durand T.C., Sergeant K., Renaut J., Planchon S., Hoffmann L., Carpin S., Label P., Morabito D., Hausman J.F. (2011). Poplar under drought: Comparison of leaf and cambial proteomic responses. J. Proteom..

[26] Rabilloud T., Jorrin-Novo J.V., Komatsu S., Weckwerth W., Wienkoop S. (2014). How to use 2D gel electrophoresis in plant proteomics. Plant Proteomics.

[27] Arc E., Galland M., Cueff G., Godin B., Lounifi I., Job D., Rajjou L. (2011). Reboot the system thanks to protein post-translational modifications and proteome diversity: How quiescent seeds restart their metabolism to prepare seedling establishment. Proteomics.

[28] Laval V., Masclaux F., Serin A., Carrière M., Roldan C., Devic M., Pont-Lezica R.F., Galaud J.-P. (2003). Seed germination is blocked in Arabidopsis putative vacuolar sorting receptor (atbp80) antisense transformants. J. Exp. Bot..

[29] Zouhar J., Muñoz A., Rojo E. (2010). Functional specialization within the vacuolar sorting receptor family: VSR1, VSR3 and VSR4 sort vacuolar storage cargo in seeds and vegetative tissues: Sorting receptors for storage proteins. Plant J..

[30] Sattler S.E. (2004). Vitamin E is essential for seed longevity and for preventing lipid peroxidation during germination. Plant Cell.

[31] Smirnoff N. (1993). The role of active oxygen in the response of plants to water deficit and desiccation. New Phytol..

[32] Fait A., Fromm H., Walter D., Galili G., Fernie A.R. (2008). Highway or byway: the metabolic role of the GABA shunt in plants. Trends Plant Sci..

[33] Michaeli S., Fait A., Lagor K., Nunes-Nesi A., Grillich N., Yellin A., Bar D., Khan M., Fernie A.R., Turano F.J. (2011). A mitochondrial GABA permease connects the GABA shunt and the TCA cycle, and is essential for normal carbon metabolism: Plant mitochondrial GABA permease. Plant J..

[34] Song H., Xu X., Wang H., Wang H., Tao Y. (2010). Exogenous γ-aminobutyric acid alleviates oxidative damage caused by aluminium and proton stresses on barley seedlings. J. Sci. Food Agric..

[35] Sgaravatti A.M., Sgarbi M.B., Testa C.G., Durigon K., Pederzolli C.D., Prestes C.C., Wyse A.T.S., Wannmacher C.M.D., Wajner M., Dutra-Filho C.S. (2007). Gamma-hydroxybutyric acid induces oxidative stress in cerebral cortex of young rats. Neurochem. Int..

[36] Ben-Izhak Monselise E., Parola A.H., Kost D. (2003). Low-frequency electromagnetic fields induce a stress effect upon higher plants, as evident by the universal stress signal, alanine. Biochem. Biophys. Res. Commun..

[37] Uemura T., Ueda T. (2014). Plant vacuolar trafficking driven by RAB and SNARE proteins. Curr. Opin. Plant Biol..

[38] Pereira-Leal J.B., Seabra M.C. (2001). Evolution of the rab family of small GTP-binding proteins. J. Mol. Biol..

[39] Cui Y., Zhao Q., Gao C., Ding Y., Zeng Y., Ueda T., Nakano A., Jiang L. (2014). Activation of the Rab7 GTPase by the MON1-CCZ1 complex is essential for PVC-to-vacuole trafficking and plant growth in Arabidopsis. Plant Cell.

[40] Ebine K., Inoue T., Ito J., Ito E., Uemura T., Goh T., Abe H., Sato K., Nakano A., Ueda T. (2014). Plant vacuolar trafficking occurs through distinctly regulated pathways. Curr. Biol..

[41] Mazel A. (2004). Induction of Salt and Osmotic Stress Tolerance by Overexpression of an Intracellular Vesicle Trafficking Protein AtRab7 (AtRabG3e). Plant Physiol..

[42] Kang H., Hwang I. (2014). Vacuolar Sorting Receptor-Mediated Trafficking of Soluble Vacuolar Proteins in Plant Cells. Plants.

[43] Yamazaki M., Shimada T., Takahashi H., Tamura K., Kondo M., Nishimura M., Hara-Nishimura I. (2008). Arabidopsis VPS35, a Retromer Component, is Required for Vacuolar Protein Sorting and Involved in Plant Growth and Leaf Senescence. Plant Cell Physiol..

[44] Kang H., Kim S.Y., Song K., Sohn E.J., Lee Y., Lee D.W., Hara-Nishimura I., Hwang I. (2012). Trafficking of vacuolar proteins: The crucial role of Arabidopsis vacuolar protein sorting 29 in recycling vacuolar sorting receptor. Plant Cell.

[45] Fuji K., Shirakawa M., Shimono Y., Kunieda T., Fukao Y., Koumoto Y., Takahashi H., Hara-Nishimura I., Shimada T. (2016). The adaptor complex AP-4 regulates vacuolar protein sorting at the trans-Golgi network by interacting with VACUOLAR SORTING RECEPTOR1. Plant Physiol..

[46] Shimada T., Yamada K., Kataoka M., Nakaune S., Koumoto Y., Kuroyanagi M., Tabata S., Kato T., Shinozaki K., Seki M. (2003). Vacuolar processing enzymes are essential for proper processing of seed storage proteins in *Arabidopsis thaliana*. J. Biol. Chem..

[47] Chae K., Gonong B.J., Kim S.-C., Kieslich C.A., Morikis D., Balasubramanian S., Lord E.M. (2010). A multifaceted study of stigma/style cysteine-rich adhesin (SCA)-like Arabidopsis lipid transfer proteins (LTPs) suggests diversified roles for these LTPs in plant growth and reproduction. J. Exp. Bot..

[48] Svenning S., Lamark T., Krause K., Johansen T. (2011). Plant NBR1 is a selective autophagy substrate and a functional hybrid of the mammalian autophagic adapters NBR1 and p62/SQSTM1. Autophagy.

[49] Shen B., Sinkevicius K.W., Selinger D.A., Tarczynski M.C. (2006). The homeobox gene GLABRA2 affects seed oil content in Arabidopsis. Plant Mol. Biol..

[50] Shi L., Katavic V., Yu Y., Kunst L., Haughn G. (2012). Arabidopsis *glabra2* mutant seeds deficient in mucilage biosynthesis produce more oil: Mucilage-deficient gl2 seeds produce more oil. Plant J..

[51] Chen M., Du X., Zhu Y., Wang Z., Hua S., Li Z., Guo W., Zhang G., Peng J., Jiang L. (2012). *Seed Fatty Acid Reducer* acts downstream of gibberellin signalling pathway to lower seed fatty acid storage in *Arabidopsis*: GA regulates seed fatty acid storage via SFARs. Plant Cell Environ..

[52] Chepyshko H., Lai C.-P., Huang L.-M., Liu J.-H., Shaw J.-F. (2012). Multifunctionality and diversity of GDSL esterase/lipase gene family in rice (*Oryza sativa* L. japonica) genome: new insights from bioinformatics analysis. BMC Genom..

[53] Durand T.C., Sergeant K., Carpin S., Label P., Morabito D., Hausman J.-F., Renaut J. (2012). Screening for changes in leaf and cambial proteome of *Populus tremula*×*P. alba* under different heat constraints. J. Plant Physiol..

[54] Eastmond P.J., Quettier A.-L., Kroon J.T.M., Craddock C., Adams N., Slabas A.R. (2010). PHOSPHATIDIC ACID PHOSPHOHYDROLASE1 and 2 eegulate phospholipid synthesis at the endoplasmic reticulum in Arabidopsis. Plant Cell.

[55] Sampedro J., Gianzo C., Iglesias N., Guitian E., Revilla G., Zarra I. (2012). AtBGAL10 is the main xyloglucan-Galactosidase in Arabidopsis, and its absence results in unusual xyloglucan subunits and growth defects. Plant Physiol..

[56] Strohmeier M., Hrmova M., Fischer M., Harvey A.J., Fincher G.B., Pleiss J. (2009). Molecular modeling of family GH16 glycoside hydrolases: Potential roles for xyloglucan transglucosylases/hydrolases in cell wall modification in the poaceae. Protein Sci..

[57] Park Y.B., Cosgrove D.J. (2012). Changes in cell wall biomechanical properties in the xyloglucan-deficient *xxt1/xxt2* mutant of Arabidopsis. Plant Physiol..

[58] Liu Y.-B., Lu S.-M., Zhang J.-F., Liu S., Lu Y.-T. (2007). A xyloglucan endotransglucosylase/hydrolase involves in growth of primary root and alters the deposition of cellulose in Arabidopsis. Planta.

[59] Vázquez-Lobo A., Roujol D., Zuñiga-Sánchez E., Albenne C., Piñero D., de Buen A.G., Jamet E. (2012). The highly conserved spermatophyte cell wall DUF642 protein family: Phylogeny and first evidence of interaction with cell wall polysaccharides *in vitro*. Mol. Phylogenet. Evol..

[60] Zúñiga-Sánchez E., Gamboa-de Buen A. (2012). The Two DUF642 At5g11420 and At4g32460-Encoded Proteins Interact in Vitro with the AtPME3 Catalytic Domain.

[61] Garza-Caligaris L.E., Avendaño-Vázquez A.O., Alvarado-López S., Zúñiga-Sánchez E., Orozco-Segovia A., Pérez-Ruíz R.V., Gamboa-deBuen A. (2012). At3g08030 transcript: A molecular marker of seed ageing. Ann. Bot..

[62] Hayashi S., Ishii T., Matsunaga T., Tominaga R., Kuromori T., Wada T., Shinozaki K., Hirayama T. (2008). The glycerophosphoryl diester phosphodiesterase-like proteins SHV3 and its homologs play important roles in cell wall organization. Plant Cell Physiol..

[63] Hong M.J., Kim D.Y., Lee T.G., Jeon W.B., Seo Y.W. (2010). Functional characterization of pectin methylesterase inhibitor (PMEI) in wheat. Genes Genet. Syst..

[64] Atmodjo M.A., Sakuragi Y., Zhu X., Burrell A.J., Mohanty S.S., Atwood J.A., Orlando R., Scheller H.V., Mohnen D. (2011). Galacturonosyltransferase (GAUT)1 and GAUT7 are the core of a plant cell wall pectin biosynthetic homogalacturonan:galacturonosyltransferase complex. Proc. Natl. Acad. Sci. USA.

[65] Rautengarten C., Usadel B., Neumetzler L., Hartmann J., Büssis D., Altmann T. (2008). A subtilisin-like serine protease essential for mucilage release from Arabidopsis seed coats. Plant J..

[66] Rajjou L., Lovigny Y., Groot S.P.C., Belghazi M., Job C., Job D. (2008). Proteome-Wide Characterization of Seed Aging in Arabidopsis: A Comparison between Artificial and Natural Aging Protocols. Plant Physiol..

[67] Bradford M. (1976). A rapid and sensitive method for the quantitation of microgram quantities of protein using the principle of protein dye binding. Anal. Biochem..

[68] Arc E., Chibani K., Grappin P., Jullien M., Godin B., Cueff G., Valot B., Balliau T., Job D., Rajjou L. (2012). Cold Stratification and Exogenous Nitrates Entail Similar Functional Proteome Adjustments during *Arabidopsis* Seed Dormancy Release. J. Proteome Res..

[69] Laemmli U.K. (1970). Cleavage of structural proteins during the assembly of the head of bacteriophage T4. Nature.

[70] Ishihama Y., Oda Y., Tabata T., Sato T., Nagasu T., Rappsilber J., Mann M. (2005). Exponentially Modified Protein Abundance Index (emPAI) for Estimation of Absolute Protein Amount in Proteomics by the Number of Sequenced Peptides per Protein. Mol. Cell Proteom..

[71] Rappsilber J. (2002). Large-Scale Proteomic Analysis of the Human Spliceosome. Genome Res..

[72] Job C. (2005). Patterns of protein oxidation in Arabidopsis seeds and during germination. Plant Physiol..

[73] Nguyen T.-P., Cueff G., Hegedus D.D., Rajjou L., Bentsink L. (2015). A role for seed storage proteins in *Arabidopsis* seed longevity. J. Exp. Bot..

[74] Xiao C., Zhang T., Zheng Y., Cosgrove D.J., Anderson C.T. (2016). Xyloglucan deficiency disrupts microtubule stability and cellulose biosynthesis in Arabidopsis, altering cell growth and morphogenesis. Plant Physiol..

[75] Wolf S. (2017). Plant cell wall signalling and receptor-like kinases. Biochem. J..

[76] Jeevan Kumar S.P., Rajendra Prasad S., Banerjee R., Thammineni C. (2015). Seed birth to death: dual functions of reactive oxygen species in seed physiology. Ann. Bot..

